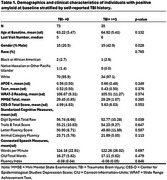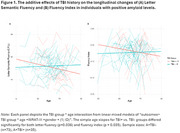# Associations Between Self‐reported History of Traumatic Brain Injury on Longitudinal Cognitive and Speech Changes Linked to Alzheimer's Disease Risk Factors

**DOI:** 10.1002/alz70857_107250

**Published:** 2025-12-25

**Authors:** Olivia Goulette, Deling He, Kristin E Basche, Rebecca E. Langhough, Tobey J. Betthauser, Bruce P Hermann, Sterling C Johnson, Kimberly D Mueller

**Affiliations:** ^1^ Department of Communication Sciences and Disorders, University of Wisconsin‐ Madison, Madison, WI, USA; ^2^ Wisconsin Alzheimer's Institute, University of Wisconsin‐Madison School of Medicine and Public Health, Madison, WI, USA; ^3^ Wisconsin Alzheimer's Disease Research Center, University of Wisconsin‐Madison School of Medicine and Public Health, Madison, WI, USA; ^4^ Department of Neurology, University of Wisconsin‐Madison School of Medicine and Public Health, Madison, WI, USA; ^5^ Wisconsin Alzheimer's Disease Research Center, School of Medicine and Public Health, University of Wisconsin‐Madison, Madison, WI, USA; ^6^ Wisconsin Alzheimer's Disease Research Center, University of Wisconsin‐Madison, School of Medicine and Public Health, Madison, WI, USA; ^7^ Department of Communication Sciences and Disorders, University of Wisconsin‐Madison, Madison, WI, USA

## Abstract

**Background:**

While both traumatic brain injury (TBI) and Alzheimer's disease (AD) are known to impair cognitive, language, and speech abilities, little to no research examines how a history of TBI interacts with AD‐related predictors (e.g., amyloid and tau pathology, familial genetic factors) to influence longitudinal changes in cognitive and language outcomes.

**Method:**

We included 308 cognitively unimpaired participants from the Wisconsin Registry for Alzheimer's Prevention (WRAP) with available amyloid and tau PET imaging, APOE ε4 genetic status, and self‐reported TBI history (TBI‐=0, TBI+ >=1). Within each of three AD risk groups (i.e., A+, T+, or APOE e4 carriers), we examined TBI history*age interactions in relationship with cognitive‐language outcomes using linear mixed‐effects models, adjusting for sex, and WRAT‐3 score [i.e., outcomes ∼ TBI group * age +sex+ WRAT‐3+ (1|ID)]. We examined standardized cognitive measures of executive functioning/set‐shifting (i.e., Digit Symbol Coding Test, Trail Making Test B Score), semantic‐phonological processing (i.e., animal category fluency, letter fluency [C, F, L]), and connected speech measures from picture description (i.e., Fluency Index, Words per Minute, Correct‐Information‐Units/total words).

**Result:**

In the A+ subset (Ntotal=108, NTBI+=35, NTBI‐=73;, Table 1), we found that the TBI+ group exhibited greater average decline in letter fluency (*p* = 0.036) and the fluency index (i.e., increases in disfluent behavior; *p* =  0.035) compared to those without TBI (Figure 1). No significant associations were observed between TBI history and longitudinal changes outcomes in the sample of tau+ (Ntotal=73, NTBI+=25, NTBI‐=48) and APOE ε4 allele carriers (Ntotal=127, NTBI+=40, NTBI‐=87).

**Conclusion:**

Our findings suggest that TBI, in combination with early AD amyloid accumulation, may accelerate communication declines, particularly manifesting as reduced letter fluency and increased speech disfluency. Presumably, these results indicate increased challenges in semantic and phonological retrieval due to the compounded impact of TBI and AD pathology in individuals who are cognitively unimpaired. Our study identifies potential early language indicators of decline; future analyses will investigate more complex TBI/biomarker relationships and additional outcomes.